# Effects of varying doses of estrogen and caudal pressure on wheel running in orchidectomized male mice

**DOI:** 10.14814/phy2.13730

**Published:** 2018-06-04

**Authors:** Brittany E. Cates, Bryce M. Dillard, Brittany R. Foster, Shawnee V. Patterson, Thomas P. Spivey, Eric B. Combs, Robert S. Bowen

**Affiliations:** ^1^ Laboratory of Applied and Exercise Endocrinology Pilgram Marpeck School of STEM Truett McConnell University Cleveland Georgia

**Keywords:** Hormones, locomotion, physical activity, sex steroids

## Abstract

Physical inactivity is a leading cause of hypokinetic diseases – obesity, heart disease, diabetes, and certain types of cancers. Increased city walkability, better access to fitness facilities, and remediation of socioeconomic barriers prove successful for limited populations within the confines of stringently controlled environments; however, these strategies fail to reverse the ever‐increasing physical inactivity epidemic on a global scale indicating the existence of other unidentified factors. These purported biological factors remain critical targets to understand the regulation of this complex phenotype. An estrogenic mechanism that incompletely or slowly adjusts physical activity levels following reintroduction of estrogenic compounds to surgically gonadectomized mice has been postulated to exist. Currently, this mechanism remains scrutinized due to concerns that elevated estrogen levels induce urinary bladder distension. The distension of the urinary bladder may mechanically disrupt physical activity, masking any physiological effects estrogen has on physical activity. The purpose of this study was to evaluate the effects of estrogen on physical activity levels while employing dose‐related strategies to alleviate distension in mice. Wheel running data were collected under normal physiological conditions, following removal of endogenous sex steroids via orchidectomy, and during estrogen replacement at various doses (0%, 10%, 50% or 100% estrogen‐containing implants) to induce varying degrees of urinary bladder distension. Wheel running distance (*P *=* *0.005) and duration (*P *=* *0.006) decreased after orchidectomy, but slowly increased following estrogen replacement. During the study, wheel running did not return to the levels observed in physiologically intact mice. Significant distension was not observed between estrogen treatment groups indicating that a slow‐responding estrogen effect exists in male mice that prevents wheel running from returning to normal levels immediately following steroid reintroduction. The limited increase in wheel running during estrogen treatment following orchidectomy is not an artifact of induced urinary bladder distension.

## Introduction

Physical inactivity is a leading cause of hypokinetic diseases in the human population (Mokdad et al. 2004). Increasing physical activity levels could substantially reduce the occurrence of diseases such as obesity, heart disease, diabetes, and certain types of cancers thereby increasing overall public health and eliminating much of the burden on the American healthcare system (Mokdad et al. 2004). Both environmental (Perusse et al. 1989; Sallis and Hovell 1990; Ferreira et al. 2007; Phongsavan et al. 2007; Wendel‐Vos et al. 2007) and biological (Lightfoot 2008, 2011; Moore‐Harrison and Lightfoot 2010; Bowen et al. 2011b) factors dictate physical activity levels, and much research has been undertaken to determine the specific roles these factors play.

Rodent wheel running activity has proven to be a good model of human physical activity and has commonly been used to explore the genetic and biological foundations for elevated physical activity (Turner et al. 2005; Leamy et al. 2008; Lightfoot et al. 2008, 2010; Knab et al. 2009, 2012; Bowen et al. 2011a, 2012). In particular, researchers have sought to elucidate the effects of the androgens and estrogens on physical activity levels in C57BL/6J mice demonstrating a significant sex steroid effect on wheel running parameters (Ogawa et al. 2003; Gorzek et al. 2007; Bowen et al. 2011a, 2012; Ibebunjo et al. 2011). Gonadectomized rodents consistently exhibit lower levels of physical activity than control animals or animals that are under physiologically normal conditions (Roy and Wade 1975; Ogawa et al. 2003; Gorzek et al. 2007; Bowen et al. 2011a, 2012; Ibebunjo et al. 2011). Previous studies have shown that estrogen replacement strategies result in varying levels of wheel running recovery – from minimal recovery (Bowen et al. 2012) to full recovery (Gorzek et al. 2007).

The mechanisms by which these effects are achieved are not fully understood. Roy and Wade (1975) demonstrated that the administration of estradiol benzoate is effective in regaining normal physical activity levels in orchidectomized male rats and testosterone's effects were mediated through estrogen‐dependent pathways. Ogawa et al. (2003) further demonstrated that the recovery response exhibited after estrogen replacement occurred through the *α* isoform of the estrogen receptor. Gorzek et al. (2007) demonstrated similar mechanisms of recovery in ovariectomized female mice. In contrast, Bowen et al. (2012) observed only slight increases in activity with the administration of estrogen; the observed increase in activity levels was not pronounced enough to reach pre‐gonadectomy levels. In particular, Bowen et al. (2012) observed minimal effects of estrogen on wheel running recovery despite administration of supraphysiological doses of the hormones, while Roy and Wade (1975) and Gorzek et al. (2007) used physiological doses of the hormone leading to the near complete recovery of wheel running. It is apparent that high estrogen doses resulted in physiologically different effects on physical activity. It has been proposed that increased levels of bladder urine may induce pressure on other surrounding tissues resulting in discomfort in mice. This claim appears to be supported by Kuroda et al. (1985), who demonstrated that high levels of circulating estrogens induced urine retention in the bladder. Furthermore, other experimental differences, including study length and estrogen administration technique, are apparent in studies that have evaluated the estrogen recovery response.

Several techniques are available to mitigate the adverse effects of estrogen dosing in mice. Caudal pressure and different concentrations of estrogen can produce a clearer picture of the estrogen recovery response and clarify the effects urinary bladder distension might have on physical activity behavior. The purpose of this study was to determine the effects of various 17*β*‐estradiol dosing schedules on wheel running activity and urinary bladder volumes. Furthermore, this study aimed to evaluate the technical utility of caudal pressure during estrogen replacement to eliminate adverse effects on the physical activity pattern in male mice.

## Methods and Materials

### Animals

Twenty‐six male C57BL/6J (Jackson Laboratory, Bar Harbor, ME) mice were used in the following experimental procedures. Mice were received at 8 weeks of age and were immediately housed individually in standard, rat‐sized cages in an animal housing room with a 12:12 hr light:dark cycle; lights turned on at 6 am daily. Each cage was equipped with a water bottle and a metal running wheel with a solid running surface. Throughout the protocol, mice were given ad libitum access to water and standard rodent chow (2018 Teklad Global 18% Protein Irradiated Rodent Diet, Harlan Laboratories, Madison, WI). This project complied with the standards of humane animal care as determined by the Truett McConnell University Institutional Animal Care and Use Committee.

### Wheel running

Running wheels (450 mm circumference; 70 mm wide running surface) were mounted to the wire cage tops and were equipped with cycling computers (BC500; Sigma Sport, Batavia, IL). Physical activity levels – distance (km), duration (min), and speed (m/min) – were quantified daily and averaged for 7 days for each experimental period. Distance and duration data were collected every 24 h at 2 pm. Average speed (m/min) was calculated by dividing distance (converted to meters) by duration. Wheel running freeness to rotate and sensor alignment were checked daily to ensure the creation of a proper daily data record. Despite best efforts, the cycling computers occasionally failed to register a complete data record for isolated 24‐h periods. On days when errors were noted, the data were excluded from analysis. Running wheels and cages were cleaned every 2 weeks for the duration of the experiment.

### Surgical orchidectomy

The present project utilized surgical orchidectomies to eliminate endogenous sex steroids from circulation. All mice received a pre‐emptive injection of Carprofen (5.0 mg/kg; Pfizer Animal Health, New York, NY) approximately 1 h before surgery. Bilateral orchidectomies were performed to remove the gonadal tissue. In brief, a small incision was made on the midline of the scrotal sac. After clipping the fascia, light pressure was applied superior to the incision to expose each testis. Both the testes and epididymis were excised and the incision was closed with a wound clip. Sham procedures were also performed in the present study and were identical to actual orchidectomies minus the excision of the gonadal tissue. All procedures were performed under light isoflurane anesthesia (2.5%) delivered by oxygen inhalation (300 mL/min). A sterile surgical field (70% ethanol and 10% betadine washes) was established and maintained throughout the procedure and all incisions were made with sterile surgical tools. A 10‐day postoperative recovery and steroid washout period were provided before reinitiating wheel running evaluation.

### Sex steroid replacement

Silastic capsules were utilized for sex steroid replacement. Capsules consisted of a 10 mm long section of Silastic tubing (Dow Corning, Midland, MI) filled with either 17*β*‐estradiol (Sigma‐Aldrich, St. Louis, MO), cholesterol (MP Biomedicals, Solon, OH), or a mixture of the two chemicals. Control animals received capsules filled with 100% cholesterol; experimental groups received capsules filled with a 10:90 mixture of 17*β*‐estradiol and cholesterol, a 50:50 mixture of 17*β*‐estradiol and cholesterol, or 100% 17*β*‐estradiol. Prepared capsules were sealed at both ends with weatherproof silicone caulk, washed in 70% ethanol, and rinsed with sterile water. Capsules containing cholesterol were stored at −20°C until use; capsules containing 100% 17*β*‐estradiol were stored at room temperature until use.

Capsules were surgically implanted between the skin and fascia on the dorsum of the animals. Briefly, a small incision was made on the dorsolateral aspect of the neck. Hemostats were used to create a pocket between the skin and muscle tissue and the capsule was placed inside the formed cavity. The skin incision was closed with a wound clip. All surgeries were performed under light isoflurane anesthesia and with sterile techniques as described above. All animals were allowed a 2‐day postprocedure recovery period to facilitate efficient release of the steroid from the capsule.

### Urinary bladder dynamics

Caudal pressure was used to eliminate urinary bladder distension induced by 17*β*‐estradiol treatment. The pressure was applied to the abdomen directly superficial to the urinary bladder to induce micturition once every 24 h immediately before the dark period between 5 and 6 pm. Expressed urine mass (mg/day) was quantified for the first 3 days of this final stage of the study on an analytical balance. The authors decided to utilize the caudal pressure technique only once per day to limit the possibility of inducing a fear response in the mice, which is well known to reduce physical activity patterns as demonstrated in open‐field testing in mice (Ogawa et al. 2003). Average daily urine mass was compared between groups. In addition, to quantify the urinary bladder contents over the course of 3 days, caudal palpation was used as a subjective measure of urinary bladder distention. The firmness and size of the urinary bladder were noted by the same technician each day prior to inducing micturition to clear the bladder of its contents. The firmness and size were not quantified, however, a noticeable pattern was evident and after a few days of caudal palpation, a blinded technician was able to determine the 17*β*‐estradiol treatment modality for each palpated mouse. The pattern was noted and used in subjective assessments of the resultant wheel running data.

### Blood collection and steroid quantification

Blood samples were collected to determine circulating sex steroid levels during the post‐orchidectomy and postimplantation periods. In order to obtain the required volume of blood/sera for successful quantification of circulating levels of 17*β*‐estradiol via enzyme‐linked immunosorbent assay (ELISA), terminal procedures were employed at a mid‐study time point. To account for the loss of animal numbers, sacrificial mice were included in each experimental group. Six mice were culled mid‐study to evaluate post‐orchidectomy sex steroid levels. All remaining mice (*n* = 20) were culled at the end of the study. Viable blood samples with volumes sufficient for triplicate measurements and with limited red cell hemolysis were obtained from 18 animals. All blood samples were obtained via cardiac puncture under heavy isoflurane anesthesia prior to full dissection to collect additional tissue and organ samples. Blood samples were allowed to clot for 45 min in a microcentrifuge tube at room temperature and were then centrifuged at 1500 × *g* for 10 min. Samples, that were not immediately centrifuged after the 45‐min incubation period, were maintained at 4°C for up to 1 h. Collected serum was snap frozen on dry ice and stored at −80°C.

Serum samples were prepared for triplicate measurement of 17*β*‐estradiol via ELISA (Cayman Chemical, Ann Arbor, MI) per manufacturer's instructions. Triplicate sample groupings that exhibit a high intra‐assay coefficient of variation (>15% as defined by commercial protocol standards) were assessed for individual samples within the triplicate grouping that diverge noticeably from the other data values in the group. These individual data points were eliminated from further analysis. Data that were below the assay's limits of detection were considered to exhibit no circulating 17*β*‐estradiol and were quantified as 0.0 pg/mL for analysis. Three triplicate groupings exhibited widely dispersed individual samples with excessive intra‐assay coefficients of variation (mean and range %CV were 116.3% and 58.5% to 152.4%, respectively). These three samples were eliminated from analysis due to the excessive intra‐assay variability. The mean intra‐assay coefficient of variation for samples above the limits of detection was 6.5%.

### Experimental procedures

**Figure 1:**
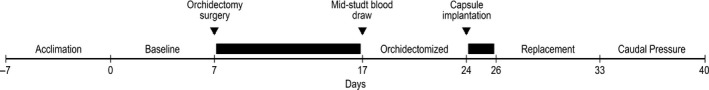
Experimental timeline to assess the effects of 17*β*‐estradiol on physical activity and induced urinary bladder distention in male mice.

The experimental timeline for the present project is displayed in Figure 1. Each wheel running observation period required 7 days. All mice were allowed a 7‐day acclimation period to stabilize the wheel running indices prior to use in any procedure. At 9 weeks of age, all mice underwent a baseline wheel running observation period. After the initial phase, mice were assigned to receive either a sham (*n* = 8) or real (*n* = 18) orchidectomy. Surgeries and a 10‐day recovery period were followed by a second wheel running observation period to evaluate the physical activity pattern under low circulating sex steroid conditions. Mice from both the sham and real orchidectomy groups (*n* = 3 per group) were euthanized for steroid quantification at the end of the second observation period. The remaining sham mice (*n* = 5) continued to act as control animals for the duration of the study. The remaining orchidectomized mice (*n* = 15) were randomly assigned into one of three experimental groups; high (100%) estrogen, moderate (50%) estrogen, low (10%) estrogen treatment groups (*n* = 5 per group). Each group received a Silastic capsule corresponding to their group assignment and underwent a brief 2‐day recovery period. Following the recovery period, wheel running was assessed to evaluate physical activity characteristics during 17*β*‐estradiol replacement. Following 17*β*‐estradiol replacement, a fourth 7‐day observation period was used to evaluate the effects of caudal pressure on wheel running in 17*β*‐estradiol‐treated mice. All remaining mice were sacrificed and blood samples were collected following this final wheel running observation period.

### Statistical Analysis

The wheel running indices (distance, duration, and speed) and sera 17*β*‐estradiol levels were analyzed by separate two‐way analysis of variance (ANOVA) calculations (group × phase). A one‐way ANOVA was utilized to compare expressed 3‐day average urine masses between each group. When significant *F* scores were found, Tukey's HSD post hoc testing was used to determine true significance. Lastly, an independent samples *t*‐test was used to compare 17*β*‐estradiol levels between treated (all low, moderate, high treatment groups combined) and nontreated (all control, orchidectomized, sham treatment groups combined) animals. An a priori alpha value of 0.05 was utilized to determined significance for all statistical comparisons.

## Results

### Distance

**Figure 2:**
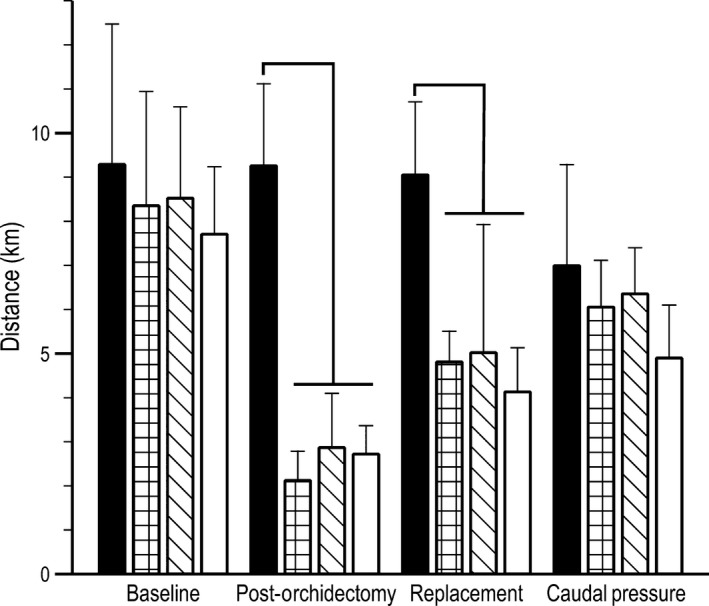
Wheel running distance (km) prior to orchidectomy (baseline), after removal of endogenous sex steroids (post‐orchidectomy), with 17*β*‐estradiol treatment (replacement), and during 17*β*‐estradiol and caudal pressure treatments to alleviate bladder distension between control (*n* = 5, black bars), low estrogen treated (*n* = 5, checkered bars), moderate estrogen treated (*n* = 5, slashed bars), and high estrogen treated (*n* = 5, open bars) mice. Brackets represent within phase statistical differences (*P *≤ 0.05).

Running distance was significantly different between some experimental groups and phases of the study (*F *=* *3.024, *P *=* *0.005, Fig. 2). Following orchidectomy, experimental animals ran significantly less than control animals. During the replacement phase, neither the low or moderate estrogen groups were significantly different from either baseline or post‐orchidectomy running levels. Distances from high estrogen mice differed significantly from the baseline phase but not levels during the post‐orchidectomy phase. During the caudal pressure phase, control, low, moderate, and high groups were not significantly different from each other; only the high estrogen group was significantly different from the control distances from the first three phases. The moderate estrogen treatment group during the caudal pressure phase of the study was significantly different from the low post‐orchidectomy group. In general, distance within experimental groups decreased dramatically after orchidectomy but gradually increased throughout the remaining phases (Fig. 2) until no statistical difference could be detected between the control and baseline data.

### Duration

**Figure 3:**
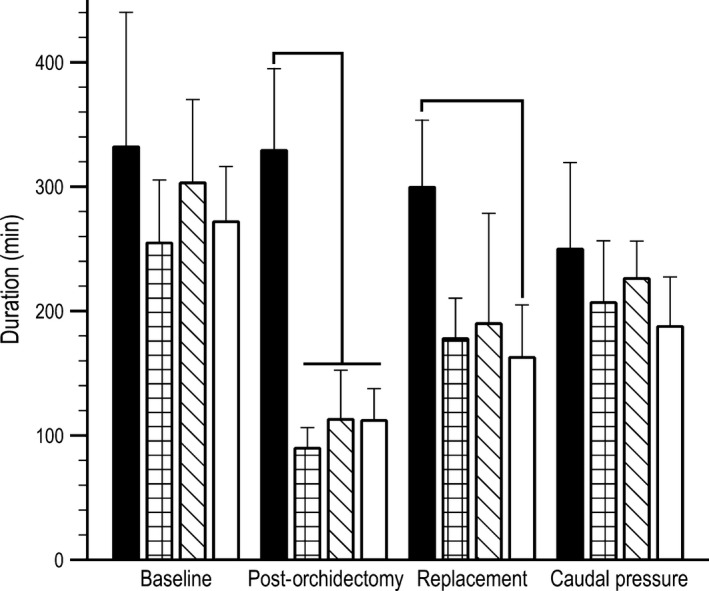
Wheel running duration (min) prior to orchidectomy (baseline), after removal of endogenous sex steroids (post‐orchidectomy), with 17*β*‐estradiol treatment (replacement), and during 17*β*‐estradiol and caudal pressure treatments to alleviate bladder distension between control (*n* = 5, black bars), low estrogen treated (*n* = 5, checkered bars), moderate estrogen treated (*n* = 5, slashed bars), and high estrogen treated (*n* = 5, open bars) mice. Brackets represent within phase statistical differences (*P *≤ 0.05).

Running duration was significantly different between some experimental groups and phases (*F *=* *2.881, *P *=* *0.006, Fig. 3). Significant differences in duration were almost identical to significant differences observed in distance. In the experimental groups, duration was significantly lower during the post‐orchidectomy phase. Wheel running duration in the experimental animals was not significantly different from either post‐orchidectomy or caudal pressure phases during replacement. Running durations during the caudal pressure phase were not significantly different from any of the other phases. Similar to distance, duration decreased following orchidectomy, but gradually increased throughout the remaining phases (Fig. 3).

### Speed

**Figure 4:**
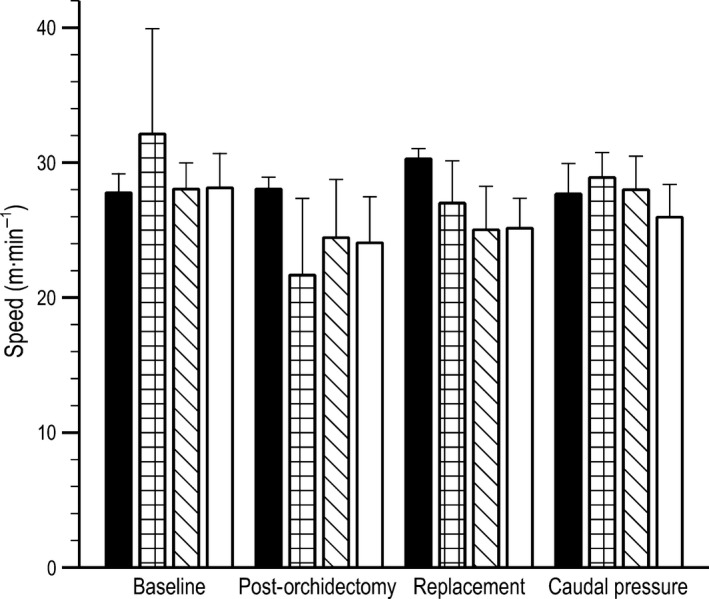
Wheel running speed (m/min) prior to orchidectomy (baseline), after removal of endogenous sex steroids (post‐orchidectomy), with 17*β*‐estradiol treatment (replacement), and during 17*β*‐estradiol and caudal pressure treatments to alleviate bladder distension between control (*n *= 5, black bars), low estrogen treated (*n* = 5, checkered bars), moderate estrogen treated (*n *= 5, slashed bars), and high estrogen treated (*n *= 5, open bars) mice. No significant differences detected (*P *≤ 0.05).

Mice maintained a consistent wheel running speed throughout the length of the study (*F *=* *1.956, *P *=* *0.06, Fig. 4). Wheel running speed averaged 27.0 ± 3.9 m/min throughout the study. Furthermore, speed did not exhibit a statistically significant relationship across treatment groups; all mice ran consistently regardless of treatment status (baseline/control = 28.9 ± 3.4, orchidectomized = 23.4 ±4.4, low treatment = 28.0 ± 2.6, moderate treatment =26.5 ± 3.1, high treatment = 25.5 ± 2.2).

### Urinary retention

**Figure 5:**
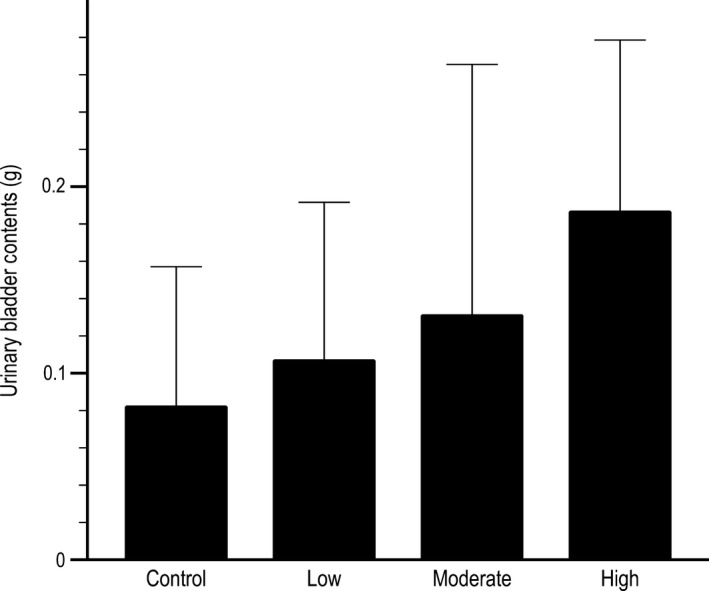
Urinary bladder contents (urine fluid mass; g) in mice treated with and without 17*β*‐estradiol. Control animals (*n* = 5) were not exposed to exogenous 17*β*‐estradiol; low estrogen treated (*n* = 5), moderate estrogen treated (*n* = 5), and high estrogen treated (*n* = 5) mice were treated with increasing amounts of 17*β*‐estradiol. No statistically significant differences were detected between the groups (*P *≤ 0.05).

Three‐day average urine output levels were not significantly different across experimental or control groups at the end of study (*F *=* *0.822, *P *=* *0.50, Fig. 5). Subjectively, urinary bladder distension noticeably increased in parallel with 17*β*‐estradiol treatment. Mice undergoing moderate and high 17*β*‐estradiol treatments exhibited larger and firmer bladders compared to the control and other treatment groups. Smaller differences in firmness and size were detectable by technicians between the lower treatment and control group.

### Sera 17β‐estradiol levels

Sera 17*β*‐estradiol levels (pg/mL) were not significantly different between control, any experimental (low, moderate, and high 17*β*‐estradiol doses), orchidectomized, or sham‐treated animals (*F *=* *2.399, *P *=* *0.12, Table 1). Lastly, 17*β*‐estradiol levels were significantly elevated (*P *=* *0.02) in animals treated with the sex steroid (low, moderate, plus high treatment groups combined; mean ± SD = 181.7 ± 153.5) compare to animals not treated with the sex steroid (control, sham, plus orchidectomized groups combined; mean ± SD = 0.0 ± 0.0; below limits of detection).

**Table 1:** Serum 17*β*‐estradiol concentrations (pg/mL)

	Mean	SD	%CV	Range	*n*
Sham	0.0	0.0	n.d.	n.d.	3
Orchidectomized	0.0	0.0	n.d.	n.d.	2
Control	0.0	0.0	n.d.	n.d.	3
Low	181.5	74.1	4.8	129.1–233.9	2
Moderate	242.8	224.1	6.6	7.7–454.1	3
High	90.4	86.5	8.1	29.2–151.5	2

Samples above the dotted line were drawn as mid‐study samples; below the dotted line, samples were drawn at the end of the study. All samples were procured from terminal blood draws.

n.d.: not determined due to assay's limit of detection.

## Discussion

The retention of urine as a byproduct of estrogen treatment is well established in the scientific literature (Kuroda et al. 1985; Kuiper et al. 1998; Levin‐Allerhand et al. 2003; Greising et al. 2011). The present study demonstrated that although the urinary system might have been affected by 17*β*‐estradiol replacement following orchidectomy in male mice, these changes were not adversely associated with observable changes to the wheel running patterns. The main finding of the present study was dosage strategies – from low to high doses of 17*β*‐estradiol – exhibited similar effects on the wheel running indices following orchidectomy. The time to recover wheel running distance and duration back to baseline levels following the loss of endogenous steroids was greater than approximately 1 week.

The effects of 17*β*‐estradiol on urinary bladder distention in this study were minimal based on the mass of urine expressed via caudal pressure during a 3‐day period at the beginning of the caudal pressure phase of this study. Subjective data based on an assessment of the urinary bladder by caudal palpation demonstrated a consistent increase in distention concomitant with increasing 17*β*‐estradiol doses. Despite the elevated distension observed with increasing levels of the steroid, wheel running characteristics did not exhibit differences between the three 17*β*‐estradiol treatment groups; running distance, duration, and speed were consistent throughout the steroid treatment phases of the study with and without caudal pressure. The lack of wheel running differences and the limited differences in urine bladder dynamics during treatment suggests that though some minor fluctuations occur in urine distention with 17*β*‐estradiol treatment these changes have limited effects on the wheel running patterns in male mice.

In their research, Roy and Wade (1975) demonstrated that 10 pg of estradiol benzoate, when administered daily in 0.1‐mL of sesame oil, increased physical activity levels of orchidectomized male rats substantially above untreated orchidectomized rats. Upon scrupulous inspection of the presented data, the invigorating effects exhibited by estrogen benzoate only becomes apparent 5 days (on days 11 and 12 of the study; based on 2‐day averaged data) after the initial introduction of the steroid. Furthermore, the estrogenic‐induced increase in physical activity did not exhibit a plateau until days 15 and 16 of the study; 8 days after initial estradiol benzoate treatment (Roy and Wade 1975). Gorzek et al. (2007) demonstrated a similar effect in female ovariectomized mice treated with estrogen pellets designed to release 3 *μ*g/day from a total available 0.18 mg of 17*β*‐estradiol. This methodology was shown to induce circulating levels (average of 20 pg/mL) in a range similar to the levels found in biologically normal female mice. Estrogen‐treated ovariectomized mice reached physical activity levels similar to physiologically intact controls during the second week following initiation of treatment. In this study, the effects of 17*β*‐estradiol were studied for 16 days following the reintroduction of 17*β*‐estradiol (day 24 of study). The wheel running distances achieved by experimental mice in low and moderate 17*β*‐estradiol treatment groups at the end of the study (days 34 through 40) were not significantly different compared to the distances ran by control mice during the same period and all treatment groups during the initial baseline evaluation period (days 1 through 7). Mice treated with the highest dosage of 17*β*‐estradiol, however, exhibited stunted recovery during both steroid replacement phases when compared to the activity patterns at baseline in all groups and the activity pattern of the control, low‐treated, and moderate‐treated animals during the experiment's final stage. Overall, the lack of a detectable difference between the aforementioned groups at various time points indicated that the estrogenic mechanism driving recovery of the physical activity patterns in male mice was not an instantaneous phenomenon.

Past research implicated a need for estrogens in an androgen‐induced physical activity regulatory pathway in rodents (Roy and Wade 1975). This mechanism was purported to utilize the aromatase enzyme complex to convert androgens – including testosterone – into estrogenic compounds prior to inducing a physiological or metabolic event that leads to changes to a rodent's wheel running pattern. The estrogen‐only paradigm was recently challenged in a series of studies by Bowen et al. (2011a, 2012). In one study (Bowen et al. 2012), supraphysiological doses of 17*β*‐estradiol and testosterone in both male and female C57BL/6J mice yielded drastically different results. When treated with testosterone, gonadectomized mice – regardless of sex – exhibited a robust recovery of their physical activity patterns to baseline levels during a 7‐day treatment period. Mice treated with estrogen exhibited a lackluster (approximately 50–60% of baseline) recovery during the same 7‐day period. In the second study, aromatase inhibiting substances were provided to male mice. The pharmacological inhibition of aromatase function decreased the physiological production of estrogens, but did not decrease wheel running vigor.

As previously stated, estrogen affects many tissues in mammals and is known to interact with the urinary bladder via the *β* isoform of the estrogen receptor (Kuiper et al. 1997). This interaction has been noted to induce significant bladder distention in mice (Kuroda et al. 1985). As such, the close anatomical proximity of the urinary bladder to the hind limbs could speculatively alter the mechanics of running during estrogen treatment, and therefore, satisfactorily explain the lackluster recovery observed during short‐term estrogen treatment and manipulation. The present study demonstrated that urinary bladder distension was only minimally different between control animals and animals treated with different dosing schedules of 17*β*‐estradiol. Furthermore, wheel running distance, duration, and speed were not significantly different within the treatment groups. The lack of a causative relationship between estrogen‐induced urinary bladder distension and wheel running dynamics in male mice suggest that typical estrogen dosing schedules do not induce confounding variability in wheel running patterns. Wheel running data from estrogen treatment studies can be interpreted directly. Lastly, the present data also suggest that estrogen treatment induces a mechanism of prolonged physical activity recovery. This prolonged mechanism is likely a different mechanism from the robust, rapid recovery mechanism exhibited with androgenic treatment.

The patterns exhibited by the highest 17*β*‐estradiol treatment strategy might indicate that extreme physiological dosing of steroids confounds the observable results during extended replacement periods and might indicate a limitation to the present data. The highest dose yielded a moderate increase in wheel running (distance = 4.90 ± 1.20 km) that remained significantly (post hoc *P *=* *0.02) lower than the control group at the beginning of the study (distance = 9.29 ± 3.18 km). The use of high dosages of 17*β*‐estradiol appears to stunt the recovery effects of 17*β*‐estradiol on wheel running patterns warranting a cautious approach to the interpretation of the data. This study is further limited by the lower numbers of animals used in this study and by the inherent difficulties associated with terminal blood collection techniques in small rodents. Both of these factors invariably reduced the power to discern significant differences between the treatment groups with regard to 17*β*‐estradiol levels as measure by ELISA. This concern, however, is significantly reduced due to the available urinary bladder content data that clearly illustrates increasing distension of the urinary bladder with increases in 17*β*‐estradiol exposure. The urinary bladder content data are a secondary bioassay that demonstrates a clear trend illustrating increased levels of estrogens in the treatment animals do not result in changes to wheel running patterns. Lastly, the use of only male C57BL/6J mice prevents crossover of these results to female C57BL/6J mice, but it is whole expected that a similar trend would be exhibited in female mice.

In conclusion, this study has shown that estrogen‐induced urinary bladder distension does not adversely inhibit wheel running recovery during steroid treatment. This study also adds additional evidence for an androgen‐induced mechanism of physical activity regulation that is separate from the postulated estrogen‐induced regulatory mechanism. Future studies should focus on the further elucidation of the overlaps and dissonances between these two purported regulatory mechanisms.

## Disclosures

The authors have nothing to disclose.
